# Soil Seed Bank in a Pre-Erosion Cereal-Grass Crop Rotation

**DOI:** 10.3390/plants11192636

**Published:** 2022-10-07

**Authors:** Regina Skuodienė, Vilija Matyžiūtė

**Affiliations:** Lithuanian Research Centre for Agriculture and Forestry Vezaiciai Branch, Gargzdu Str. 29, Vezaiciai LT-96216, Klaipeda District, Lithuania

**Keywords:** hilly relief, seed bank changes, vertical distribution, seed and plant species, segetal plants, floristic similarity

## Abstract

Soil erosion by water is a serious environmental problem. It is very important to form agrophytocenosis, which is productive on hilly terrain and could effectively protect the soil from erosion. The aim of the study was to examine the impact of a hilly relief on the changes of seeds in a soil of pre-erosion cereal-grass crop rotation. The study included different parts of the hill (summit, midslope, and footslope). The southern exposition slope’s soil was slightly eroded Eutric Retisol (loamic) (RT-eu.lo), and the steepness of the slope was 9–11°. The seed bank was investigated at the depths of 0–5 and 5–15 cm. The number of seeds in the soil seed bank during autumn was 20.4% higher than in the spring. The highest number of seeds in the autumn was determined on the summit (40.6 thousand seeds m^–2^). The highest count during the spring was determined on the midslope of the hill (36.4 thousand seeds m^–2^). In general, in the soil seed bank of the midslope of the hill, the number of seeds was by 7.8% and 42.4% higher compared to the summit and footslope parts. The highest seed reserve was found in the soil at a 0–5 cm depth (59.4% of the total seed number).

## 1. Introduction

The weed seed bank is the reserve of the viable weed seeds that are present on the soil surface and scattered in the soil profile. It consists of both new weed seeds recently shed and older seeds that have persisted in the soil for several years [1]. The weed incidence is largely based on the contamination of soil by weed seeds, the biological properties of the cultivated plant, and its overshadowing abilities [2,3]. The alterations of weed communities and the contamination of soil by weed seeds appears because of differences in soil pH, pedological aspects, and crop management [4]. The seed bank extent and a high number of species influence the dynamics of vegetation [5]. Soil tillage also has a significant influence on the dynamics of seeds [6,7]. The highest seed reserves are found in the upper soil layer (0–5 cm) [8,9] and gradually decrease moving down the soil profile [10].

Kumar et al. [11]. determined that under reduced tillage systems such as chisel ploughing, approximately 80–90% of the weed seeds are distributed in the top ten centimetres of the soil profile. In no-till fields, the majority of weed seeds remain at or near the soil surface. 

Tillage systems cause changes in the density and composition of soil weed seed bank [12,13], especially in weed seed composition in the upper 0–15 cm soil layer [14]. Studies showed that the highest number of weed seed species was found in the treatments with reduced and no-tillage treatments in a soil layer of 0–5 cm. In deeper soil layers (5–10, 10–20 cm), no differences in weed seed species number were found [15]. The species richness decreased with increases in soil depth [16]. Although seed banks and the resulting weed populations are composed of many species, a few dominant species generally comprise 70–90% of the total seed bank [8].

About 14% of Lithuania‘s agricultural land is eroded, but this number is higher in a hilly relief, where it reaches up to 25–53% [17]. Due to uneven soil erosion in select parts of the hill, different conditions in the hilly topography become present for the plant development, including differences in soil humidity, acidity, richness in nutrients, humus, and others [18]. It is believed that the microclimatic conditions of hilly relief influence crop weediness and soil seed bank. The aim of the study was to examine the impact of a hilly relief on the changes of seeds in a soil of pre-erosion cereal-grass crop rotation.

## 2. Results

### 2.1. Soil Seed Bank and Vertical Profile 

**Figure 1:**
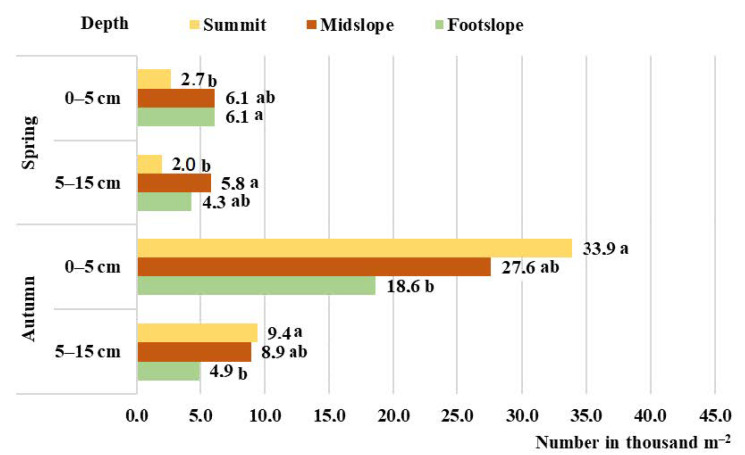
The number of seeds in a pre-erosion cereal-grass crop rotation soil in 2020. Letters “a” and “b” indicate significant (*p* < 0.05) differences between the means.

**Figure 2:**
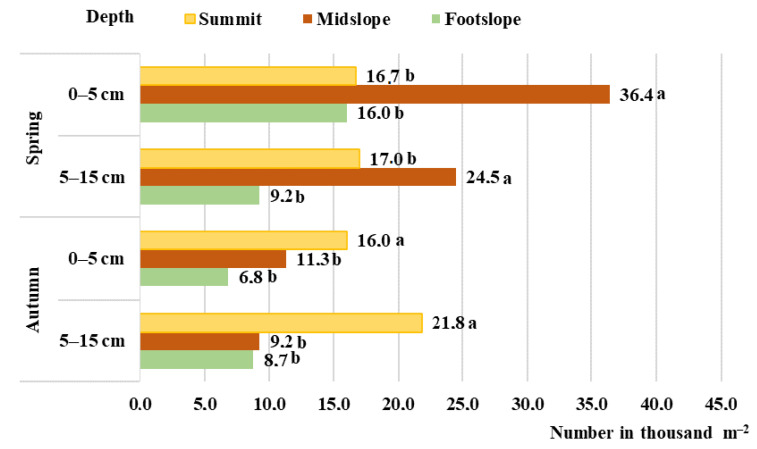
The number of seeds in a pre-erosion cereal-grass crop rotation soil in 2021. Letters “a” and “b” indicate significant (*p* < 0.05) differences between the means.

In the spring of 2020 under the conditions of hilly relief in the soil seed bank at the depth of 0–15 cm (*p* = ≤ 0.01), the smallest number of seeds (4.8 thousand seeds m^–2^) was determined in the soil of the summit of the hill, while the highest (12.0 thousand seeds m^–2^) number of seeds was found in the midslope part of the hill (Figure 1). In 2021, the smallest number of seeds (25.4 thousand seeds m^–2^) was determined in the footslope of the hill, (*p* = ≤ 0.01), while the highest (60.9 thousand seeds m^–2^) number of seeds was found in the midslope part of the hill (Figure 2). 

Analysing the soil seed bank in the autumn of 2020 and 2021, a similar tendency was found. Significantly (*p* = ≤ 0.01), the smallest (23.6 and 15.5 thousand seeds m^–2^) number of seeds was determined in the soil of the summit of the hill, while the highest (43.4 and 37.9 thousand seeds m^–2^) number of seeds was found in the midslope part of the hill, respectively, in 2020 and 2021 (Figure 1 and Figure 2). 

The seed number in the upper and lower arable soil layers differed. In spring, in the seed bank of the upper (0–5 cm depth) soil layer, the seed number ranged from 53.1 to 60.9% of the total seed number, while in autumn, the number of seeds at the same depth was determined to be higher by 1.0–17.9%, and this formed from 60.2 to 65.3% of the total seed number. In all examined cases, the highest number of weeds was determined in the footslope part of the hill, while the smallest was in the summit (Figure 1 and Figure 2).

### 2.2. Soil Seed Bank Composition and Comparison with Segetal Plant (Weeds) in Crops

**Figure 3:**
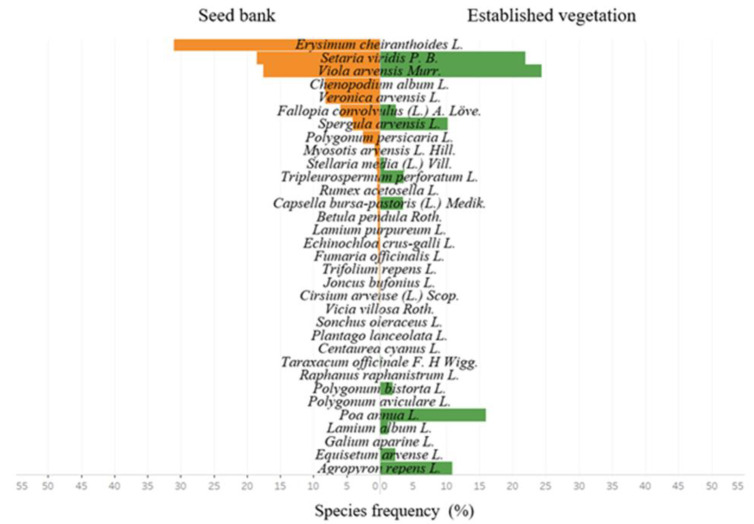
Percentage frequency of the species content of the seed bank in spring (left) and established vegetation in first assessment (right) in 2020–2021.

**Figure 4:**
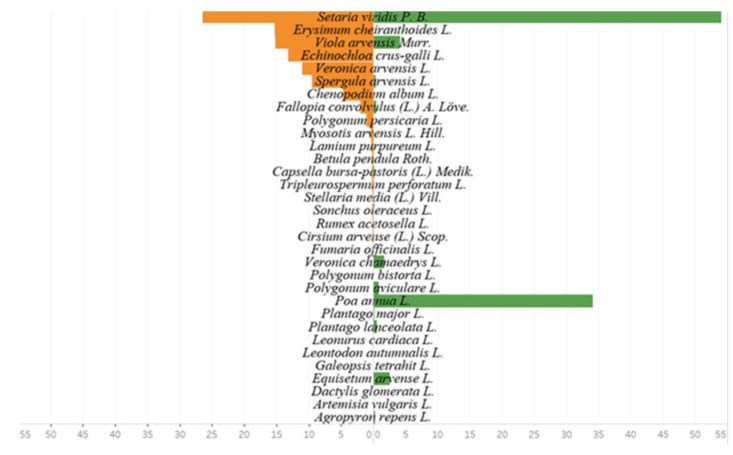
Percentage frequency of the species content of the seed bank in autumn (left) and established vegetation in the second assessment (right) in 2020–2021.

During the research, 22 seed species were found in the soil seed bank in 2020 and 21 seed species were found in 2021. The species of dicotyledonous plants formed 95.5 and 90.5% of all founded species, respectively, in 2020 and 2021 (Figure 3 and Figure 4).

According to the data of the dispersive analysis, hill parts had the essential influence on the number of species of segetal plants in the arable soil layer. In all the study years, in the footslope of the hill, the number of discovered seed species was significantly higher (11–12 species) compared to the summit and midslope. 

In the spring of 2020, the seeds of *Chenopodium album* L. and *Viola arvensis* Murr. were found in all parts of the hill, and they comprised 64.3, 74.8, and 52.5% of the total seed number in the soil seed bank, respectively, in the summit, midslope, and footslope of the hill.

In the autumn (2020), the seeds of *Setaria viridis* P. B. were mostly found in the summit (79.8%); the seeds of *Erysimum cheiranthoides* L. (40.0%) and *Veronica arvensis* L. (30.0%) in the midslope; and the seeds of *Viola arvensis* (27.1%), *Veronica arvensis* (20.9%), and *Erysimum cheiranthoides* (20.9%) in the footslope of the hill. The seeds of *Chenopodium album* and *Viola arvensis* comprised only 13.4, 13.6, and 31.2% of the total seed number in the seed bank, respectively, in the summit, midslope, and footslope of the hill.

In the spring of 2021, the seeds of *Setaria viridis* were mostly found in the summit (58.1%); the seeds of *Erysimum cheiranthoides* (60.0%) in the midslope; and the seeds of *Erysimum cheiranthoides* (32.1%), *Viola arvensis* (16.3%), and *Veronica arvensis* (12.5%) in the footslope of the hill. 

In the autumn (2021), the seeds of *Echinochloa crus-galli* L. were found in the summit of the hill (55.2%), while the seeds of *Viola arvensis* were found in the midslope and footslope parts (24.3 and 26.6%).

The seeds of *Chenopodium album, Viola arvensis, Fallopia convolvulus* (L.) A. Löve, *Setaria viridis*, and *Veronica arvensis* were found in all parts of the hill. The seeds of *Betula pendula* Roth. and *Spergula arvensis* L. were found only in the summit, the seeds of *Myosotis arvensis* L. Hill were found only in the midslope, and the seeds of *Lamium purpureum* L. were found only in the footslope part of the hill.

The average data of 2020 and 2021 showed that the soil seed bank in spring consisted of 24 species of plant seeds, while 20 segetal species were found in crops during the first assessment. Comparing the species composition of the seed bank and weed species composition in crops in spring, 11 common plant species were determined. *Viola arvensis* (17.6% in the seed bank; 24% in crops); *Setaria viridis* (18.5% in the seed bank; 21.9% in crops); and *Spergula arvensis* (4.1% in the seed bank; 10.3% in crops) formed the majority (Figure 3). In the soil seed bank in spring, the seeds of *Erysimum cheiranthoides* comprised a third part (30.9%). However, during the first assessment this species was not determined in crops.

The soil seed bank in autumn consisted of 19 species of seeds, while 21 segetal species were found in crops. Comparing the species composition of the soil seed bank and the weed species composition in crops during the second assessment, eight common plant species were determined. The most dominant species were *Viola arvensis* (15.2% in the seed bank; 4.1% in crops) and *Setaria viridis* (26.5% in the seed bank; 54.1% in crops) (Figure 4). In autumn, *Setaria viridis* (54.1%) and *Poa annua* L. (34.1%) overshadowed crops the most. *Setaria viridis* (26.5%), *Viola arvensis* Murr. (15.2%), and *Echinochloa crus-galli* (13.2%) composed a great part of the soil seed bank.

The research showed that in the footslope soil of the hill, an average of 1.2 to 1.7 times more seed species were found compared to other parts of the hill. In most cases, the closest in species composition (Cs = 0.71–0.89) was the soil seed bank in the midslope and footslope of the hill, while the least similar (Cs = 0.44–0.74) was the soil seed bank in the summit and footslope parts (Table 1). 

Edaphic factors such as soil moisture, acidity, and nutrition are very important to the growth and development of crops and segetal plants. It was found that 43.3%, 48.4%, and 48.5% of all vascular plants of the cereal-grass crop rotation in the soil of the summit, the midslope, and the footslope of the hill, respectively, were mesophyte species. Soil acidity was not a determining factor for the higher part (57.1%) of plants. The higher part of mesotrophic and eutromesotriphic plants were found in the footslope part (29.7% and 34.7%, respectively). 

## 3. Discussion

### 3.1. Soil Seed Bank and Vertical Profile

The weed soil seed bank is of interest in agroecosystems as a major source of weed infestation in fields and as a reservoir of plant and seed-feeder diversity [19]. The number of seeds in the soil is dynamic. Annually, the part of the seeds that enter the soil become dormant, and the other part of the seeds decay [20]. Although sampling time may depend on research goals for vegetation types with a winter standstill period, late autumn sampling is suggested for detecting the entire soil seed bank, whilst late spring sampling is recommended for the examination of its persistent part [21].

According to Butkevičienė et al. [22], the research data show that 20.2 to 71.4 thousand weed seeds can be found in 1 m^2^ of the cultivated soil in the depth of 0–20 cm. In 1 m^2^ of the uncultivated soil (natural meadow) in the depth of 0–15 cm, 4.3 thousand weed seeds were found [23]. The average data of 2020 and 2021 show that in the depth of 0–15 cm of the pre-erosion cereal-grass crop rotation soil, the number of seeds reached 29.9, 32.5, and 18.7 thousand, respectively, in the summit, the midslope, and the footslope parts of the hill. Considering the hilly relief conditions, the number of seeds in the soil seed bank during autumn was 20.4% higher than in spring. The highest number of seeds in autumn was determined in the summit (40.6 thousand seeds m^–2^).

The influence of meteorological conditions (precipitation) on soil contamination by plant seeds was observed in our study. Due to variations in humidity as well as the impact of soil biota [24,25] after the autumn-winter period in spring, the seed bank was 2.1 times smaller in the summit of the hill compared to autumn. The seed number decreases together with increasing precipitation, thus, showing strong negative correlations: y = 260.068 − 0.004x, r = −0.817, *p* ≤ 0.05. The highest number of seeds during spring was determined on the midslope of the hill (36.4 thousand seeds m^–2^). This could have been caused by seed transportation due to precipitation [26].

The number of weed seeds is very diverse in both the horizontal as well as vertical directions. The redistribution of fallen seeds is mostly caused by natural processes and soil management [6,27,28]. The majority of seeds are in rest; therefore, the viable seeds can be accumulated in the soil for several or even more than ten years [29]. Only the seeds that are present in the upper soil layers can germinate, while the seeds that are present in the deeper soil layers only make the potential reserves [30]. Changes in land-use and management practices influence the distribution of seeds in the soil and the established vegetation [31]. By reducing soil tillage, the seeds of segetal plants concentrate in the upper soil layer, unlike in the soil with deep ploughing applied [32]. In our research under the conditions of hilly relief, reduced soil cultivation was applied. In this way, the number of seeds in the depth of 0–5 cm increased (from 56.7 to 61.2 %) in the downslope direction. 

The literature showed that in reduced soil tillage treatments the number of weed seeds was 1.6 times greater than in conventional ploughing plots. The type of tillage strongly influenced the vertical seed placement in the soil. The annual application of the shallow ploughless tillage mostly influenced soil contamination with weed seeds (72.3% of the total seed number) at the depth of 0–10 cm. At the same depth, 61.1% of weed seeds were found after the use of shallow ploughing, and 43.7% of weed seeds of the total seed number were found after the use of conventional ploughing [33]. Feledyn-Szewczyk et al. [13] also confirm that under direct sowing, most weed seeds were accumulated in the top soil layer of 0–5 cm, while in the ploughing system, most weed seeds were found in deeper layers: 5–10 and 10–20 cm. Reduced tillage allows the bulk of the seed produced in the previous year to remain in the most superficial layer of soil, leading to its extensive germination and emergence in subsequent years [34].

### 3.2. Soil Seed Bank Composition and Comparison with Segetal Plants in Crops

Researchers identify different numbers of plant species in the soil. A high variation of plant species (38 and 53) was found in Belgium and France [35,36]; other studies recorded from 5 to 19 species [15,19,32,37,38], and in our research, 25 plant species were identified. 

The composition of the soil seed bank depends on the plant communities appearing in a particular area at present and in the past, as well as on the biological properties of plants [31]. The plants that germinated, grew, and developed on the hilly topography had different conditions [39]. While the amount of physical clay and mud increased on the lower parts of the slope, the humidity reserves increased, as well as the amount of humus and nutrients, and the acidity also decreased (Table 2). 

It is usually indicated that only a few species compose the highest part of the soil seed bank. The authors indicated that soils are mostly contaminated by the seeds of *Chenopodium album* L. [22,40,41], whose annual decline rates were about 14–41% [42]. In our research, the seeds of *Chenopodium album* prevailed in the soil seed bank in the spring of 2020, while in the autumn (2020) and in the spring and autumn of 2021, they comprised from 2.5 to 10.8% of the whole seed bank. *Viola arvensis* seeds were also found in all the research treatments. These results are in accordance with previous studies, which indicated that the seed bank is composed of the few dominant weed species [15,23,43,44]. This process is determined by their biological properties—a high number of seeds (*Chenopodium album*) [45] and less sensitivity to herbicides (*Viola arvensis*) [46]. The results of the research confirm that, due to limited viability, most of the segetal plants’ species do not compose persistent seed banks. The literature indicates that they only maintain a transient seed bank and, therefore, were absent from the seed bank at the time of sampling. On the other hand, it cannot be excluded that other species were not detected due to a lack of appropriate conditions for germination [47].

Composition of the seed bank can also be assessed as the indicator of crop management success and failure. Sadrabadi Haghighi et al. [41] showed that although crop type in a single year significantly affected the weed functional group and cover of individual species, it did not change the weed functional group and composition in the seed bank in the following year. 

In the crops of spring barley and spring wheat, agricultural and segetal plants usually begin to germinate together. As the seeds of segetal plants are mostly bloated and the weather in the Baltic States after sowing often becomes cold or dry, agricultural plants stop germinating and segetal plants often germinate earlier. Management intensity therefore impacts directly on many aspects of the seed bank, establishes vegetation relationships, and controls established vegetation, partly through seed bank dynamics involving both temporal and spatial dispersal [48].

In both study years, the similarity of segetal plants’ species in crops and seed bank species composition was determined to be minor (Cs = 0.10–0.48). Harbuch [49] indicates that the species of segetal plants in crops are not always detected in the seed bank and not all species of the seed bank can be found in the overground community. However, the samples of the soil seed bank that were collected in the autumn can be well related to the community of segetal plants in the crops of the growing season. 

A low correlation coefficient between the number of segetal plants in the field and the seed number in soil shows that high plant densities did not automatically increase the seed bank. Teasdale et al. [50] observed that a high weed cover and corresponding seed production normally lead to an increase in the seed bank when the initial seed number in soil is low. However, when the initial seed bank is high, seed losses can exceed the gains and may reduce the seed bank in the following spring. This may be a major reason why a close relationship between the field vegetation and the seed bank only occurred when plant numbers fell below 50 m^–2^ [51].

## 4. Materials and Methods

### 4.1. Site and Soil Description and Experimental Design

The experiment was carried out at the Vėžaičiai Branch of the Lithuanian Research Centre for Agriculture and Forestry, on the midslope soil of Žemaičiai Highland covered by different anti-erosion agrophytocenoses [52] in Kaltinėnai (lat. 55°57′ N, long. 22°48′ E, 185.0 m a. s. l.). The steepness of the slope was 9–11°. The soil of the southern exposition slope was slightly eroded Eutric Retisol (loamic), according to WRB [53], with a texture of sandy loam. The agrochemical and physical properties of the soil are presented in Table 2.

The research slope was 65 metres in length and the strip was 3.2 metres in width. The study included different parts of the hill (summit, midslope and footslope).

The six-course crop rotation consisted of *Hordeum vulgare* L. with under-sown perennial grasses: *Trifolium pratense* L. 80% and *Phleum pratense* L. 20% (2016), perennial grasses (2017), perennial grasses (2018), *Triticum aestivum* L. (winter crop) (2019), *Hordeum vulgare* (2020), and *Triticum aestivum* (spring crop) (2021). 

Soil samples were taken from the pre-erosion cereal-grass crop rotation in which crops of *Hordeum vulgare* (2020) and *Triticum aestivum* (2021) had been grown. 

All treatments were equally fertilised with granular mineral fertilisers (background fertilisation). The rate of fertiliser N_60_P_60_K_60_ was applied for spring barley as well as for spring wheat. Reduced soil cultivation (12–15 cm depth) was applied in spring. The stubble breaking was performed first, followed by cultivation.. During winter, stubble was left on the slope to secure its soil from erosion. Before sowing, the grains of spring wheat and spring barley were treated with Kinto (a.i. triticonazole + prochloraze) at a rate of 2 L t^–1^. Plant protection products (pesticides) were used as well: in 2020, at BBCH 32–MCPA (a.i. MCPA 750 g L) 30 mL and Arrat (a.i. dicamba-sodio + tritosulfuron) 10 mL; in 2021, BBCH 33–Trimmer 50 SG (a.i tribenuron metil) 15 g ha^–1^ and Elegant 2FD (a.i. florasulam 6.25 g L + 2,4-D 300 g L) 0,6 L ha^–1^.

### 4.2. Methods of Analysis

To assess the impact of the hill slope on soil contamination by seeds, the seed bank was investigated at the depths of 0–5 and 5–15 cm. The seed bank was estimated from soil samples taken in the spring (April) and autumn (September) of 2020 and 2021. In each model plot, 2 kg of soil from 20 positions was collected using a hand auger. The soil was dried out. In total, five 100 g samples were removed from 2 kg of soil sample and weighed. Later, the soil samples were wet sieved through a 0.25 mm sieve until all the contents of the soil were washed out. The remaining mineral part of the soil was separated from the organic part and seeds using the saturated salt solution. The seeds were identified using binoculars with 8.75× magnification. Seed viability was determined by “destructive crushing” using forceps [54]. The number of viable seeds (A) was recalculated to thousands of seeds per m^2^:

A = n × h × p × 100,(1)

where A is the number of viable seeds, seeds, m^2^; n is the counted number of viable seeds in the soil sample; h is the depth of the plough layer, cm; and p is the soil bulk density, g cm^3^.

The Latin names of seed species are presented using the book “Fruits and seeds of Lithuanian plants” [55].

The crop weed analysis was carried out in stationary 0.25 m^–2^ lots in six different parts of the field. The weediness was evaluated in 2020 in spring barley during BBCH 58 and BBCH 86 stages. In 2021, the weediness was evaluated in the spring barley crop during the stages of BBCH 32 and BBCH 87. During both evaluations, the weed type composition was determined. The number of weeds was recounted by the unit of m^–2^.

To express the floristic similarity of phytocenoses or the similarity of the seed bank and actual vegetation, the coefficient of Sörensen (Cs) was used:

Cs = 2w/(A + B),(2)

where w is the number of common species in both situations, A is the number of species in one of two comparable situations, and B is the number of species in another situation.

Chemical analyses were carried out at the Chemical Research Laboratory of the Institute of Agriculture, Lithuanian Research Centre for Agriculture and Forestry. Before establishing the experiment, soil agrochemical characteristics were determined from the samples taken from the depths of 0–5 and 5–15 cm. Soil acidity (pH) was measured by the potentiometric method in the extraction of 1 M of KCl (pH_KCl_), according to International standard ISO 10390:2005 (soil quality—determination of pH). In the soil, mobile P_2_O_5_ and K_2_O were determined using the Egner–Riehm–Domingo (AL) method (LVP D-07:2016), total nitrogen (N_tot_) content by the Kjeldahl method, and organic carbon (C_org_) by the Dumas dry combustion method. Soil bulk density was determined with a 100 cm^3^ cylindrical drill by the Kachinsky method. Soil texture was determined by the Fere triangle (FAO recommended method), according to the percentage of sand, silt, and clay fractions in the graphical diagram.

### 4.3. Agrometeorological Conditions

**Figure 5:**
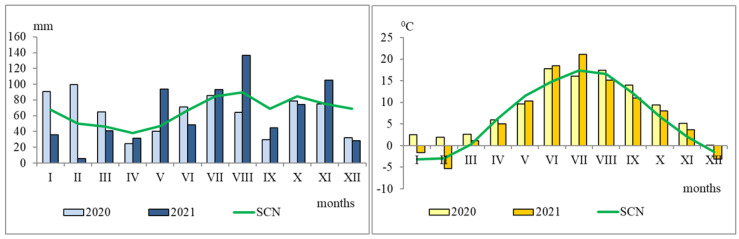
The average daily air temperature (°C) and precipitation (mm) during the study period (2020–2021). SCN—standard climate norm.

In 2020, the amount of precipitation throughout the year reached 92.6% of the Standard Climate Norm SCN (Figure 5). During the growing season, the amount of precipitation was by 18.2% lower and in the summertime by 8.8% lower compared to the SCN. Soil moisture at a 0–15 cm depth in the summit of the hill was lower (by 11.9–16.1%) than the optimal moisture for the plants (Table 2). 

In 2021, the amount of precipitation was by 3.4% higher compared to the SCN (Figure 5). During the growing season and summertime, the amount of precipitation was by 8.6 and 15.0% higher compared to the SCN. The soil in the midslope and footslope of the hill was on average by 14.0–25.2% more humid compared to the summit.

### 4.4. Statistical Analysis

The significance of the differences between the means was determined according to the Fisher’s protected Least Significant Difference (LSD) at a 0.05 probability level. The experimental data were subjected to the analysis of variance (ANOVA) [56]. The actual data of the seed bank were transformed (Sqr(x + 1)).

## 5. Conclusions

In spring (the beginning of plant vegetation), the seed number in the soil of the midslope of the hill was determined to be higher by 1.9 and 2.0 times compared to the summit and footslope of the hill. In autumn (the end of plant vegetation), the seed number in the soil of the summit of the hill was determined to be higher by 1.4 and 2.1 times compared to the midslope and footslope of the hill. 

The number of seeds at the soil depth of 0–5 and 5–15 cm changed depending on the relief. In the top-soil layer, the number of seeds increased in the downslope direction by 56.7%, 60.4%, and 61.2%, respectively, in the summit, midslope, and footslope parts of the hill. 

The seeds of *Chenopodium album, Viola arvensis, Fallopia convolvulus, Setaria viridis*, and *Veronica arvensis* were found in all parts of the hill. The seeds of *Betula pendula* and *Spergula arvensis* were found only in the summit, the seeds of *Myosotis arvensis* were found only in the midslope, and the seeds of *Lamium purpureum* were found only in the footslope part of the hill.

The closest in species composition (Cs = 0.71–0.89) was the soil seed bank in the midslope and footslope of the hill, while the least similar (Cs = 0.44–0.74) was the soil seed bank in the summit and footslope parts. The floristic similarity of segetal plants’ species in crops and seed bank species composition in all parts of the hill was determined to be minor (Cs = 0.10–0.48).

## Figures and Tables

**Table 1:** Floristic similarity coefficients of Sörensen (Cs).

Parts of the Hill	Floristic Similarity Between Parts of the Hill			
	Spring		Autumn	
	2020	2021	2020	2021
Summit and midslope	0.70	0.75	0.83	0.70
Summit and footslope	0.44	0.71	0.74	0.67
Midslope and footslope	0.71	0.77	0.74	0.89
	Floristic similarity between autumn in 2020 and spring in 2021			
	summit	midslope		footslope
Summit	0.75			
Midslope		0.91		
Footslope				0.79

Due to recurring difficulties in accomplishing crop management tasks in time, *Setaria viridis* spread in crops in 2020. Therefore, the species composition of the seed bank in the spring of 2021 was especially similar to the seed bank in the autumn of 2020 (in the summit—Cs = 0.75, in the midslope—Cs = 0.91 and in the footslope—Cs = 0.79).

**Table 2:** Agrochemical and physical properties of the arable (0–15 cm) soil layer (2020).

Soil Properties	Part of the Hill					
	Summit		Midslope		Footslope	
	0–5 cm	5–15 cm	0–5 cm	5–15 cm	0–5 cm	5–15 cm
Soil acidity (pH_KCl_)	5.6	5.4	5.3	5.1	5.1	5.1
Mobile P_2_O_5_ (mg/kg)	192	201	165	168	149	148
Mobile K_2_O (mg/kg)	209	112	198	98	223	107
Total N (%)	0.078	0.077	0.097	0.096	0.106	0.101
Organic C (%)	0.9	0.8	1.1	1.0	1.1	1.0
Soil texture	sandy loam	sandy loam	sandy loam	sandy loam	sandy loam	sandy loam
Sand (%)	79.2	77.4	75.6	75.9	73.0	69.7
Silt (%)	15.5	17.8	19.0	18.8	19.0	22.5
Clay (%)	5.3	4.8	5.4	5.3	8.0	7.8
Soil moisture ^1^ (%)	12.1–16.1	11.9–14.7	15.4–21.2	14.1–19.4	17.8–21.8	16.9–20.6

^1^—Min–max. values during the growing season.

## Data Availability

Not applicable.
